# Retrospective, real‐life study of venetoclax plus azacitidine or low‐dose cytarabine in French patients with acute myeloid leukemia ineligible for intensive chemotherapy

**DOI:** 10.1002/cam4.5459

**Published:** 2022-12-08

**Authors:** Louise Laloi, Natacha Chaumard Billotey, Pierre‐Yves Dumas, Franciane Paul, Alban Villate, Célestine Simand, Luc Fornecker, Florent Puisset, Sarah Bertoli, Marion Boissard Simonet, Kamel Laribi, Dyhia Houyou, Alberto Santagostino, Claire Michel, Gabrielle Roth Guepin, Elodie Guerineau, Reza Tabrizi, Mathilde Hunault, Aurélien Giltat, Eléonore Kaphan, Claude Bulabois, Elodie Cartet, Clément Rocher, Florence Lachenal, Stéphane Morisset, Christian Récher, Arnaud Pigneux, Amine Belhabri, Mauricette Michallet, Anne‐Sophie Michallet

**Affiliations:** ^1^ Department of Pharmacy Centre Léon Bérard Lyon France; ^2^ Department of Hematology University Hospital of Bordeaux Bordeaux France; ^3^ Department of Hematology University Hospital of Montpellier Montpellier France; ^4^ Department of Hematology University Hospital of Tours Tours France; ^5^ Department of Hematology University Hospital of Strasbourg Strasbourg France; ^6^ Department of Pharmacy Institut Universitaire du Cancer Oncopole Toulouse France; ^7^ Department of Hematology Institut Universitaire du Cancer Oncopole Toulouse France; ^8^ Department of Hematology University Hospital of Besançon Besançon France; ^9^ Department of Hematology Hospital of Le Mans Le Mans France; ^10^ Department of Clinical Research Hospital of Troyes Troyes France; ^11^ Department of Hematology Hospital of Troyes Troyes France; ^12^ Department of Hematology University Hospital of Nancy Nancy France; ^13^ Department of Clinical Research Hospital of Mont de Marsan Mont de Marsan France; ^14^ Department of Hematology Hospital of Mont de Marsan Mont de Marsan France; ^15^ Department of Hematology University Hospital of Angers Angers France; ^16^ Department of Hematology University Hospital of Grenoble Grenoble France; ^17^ Department of Pharmacy Hospital of Bourgoin‐Jallieu Bourgoin‐Jallieu France; ^18^ Department of Hematology Hospital of Bourgoin‐Jallieu Bourgoin‐Jallieu France; ^19^ Independent Biostatistician Pérouges France; ^20^ Department of Hematology and Medical Oncology Centre Léon Bérard Lyon France

**Keywords:** acute myeloid leukemia, azacitidine, low‐dose cytarabine, venetoclax

## Abstract

**Background:**

Recently, the combination of venetoclax plus a hypomethylating agent (HMA; azacitidine ordecitabine) or low‐dose cytarabine (LDAC) showed promise in Phase III trials in previously untreated AML. In France at the time of this study, venetoclax was not yet approved for AML and there were therefore no formal usage recommendations. Here we report the first study in a French cohort that assessed venetoclax in combination with existing treatments for AML under real‐life conditions.

**Method:**

This retrospective, real‐life study collected data on venetoclax use and management in a French cohort with acute myeloid leukemia (AML) ineligible for intensive chemotherapy.

**Result:**

Of 118 patients, 81 were in second line/beyond (71.6% also hypomethylating agent [HMA]; 23.5% lowdose cytarabine [LDAC]) and 37 in first line. For venetoclax initiation, 57.3% underwent ramp up and 74.6% were hospitalized. Median venetoclax duration was 2.5 months (range 0.03‐16.2). With all treatment lines and regimens, most common grade 3/4 adverse events were hematologic (overall 96.4% of patients) and infections (57.1%). Dosage adjustments for drug interactions and safety varied between centers. In second‐line/beyond, median progression‐free survival was 4.0 months (95% confidence interval [CI] 2.7‐12.8) with venetoclax‐HMA and 3.4 months (1.3‐8.9) with venetoclax‐LDAC; overall response rate was 51.9% and 41.2%, respectively. Thus, we showed that venetoclax‐based treatment yields promising findings in patients with AML, but to address treatment complexity, practice harmonization is needed.

## Graphical abstract

Real life study of venetoclax treatment line and concomitant treatment with a hypomethylating agent (HMA)or low‐dose cytarabine (LDAC) in LAM. Venetoclax base treatment yields promising finding in AML patients ineligible to intensive chemotherapy. Practice harmonization could be future improvments.

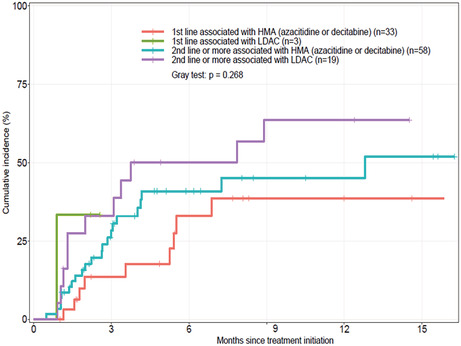

## 1 INTRODUCTION

Acute myeloid leukemia (AML) mainly affects older adults,^1^ who often present with comorbidities and are ineligible for intensive chemotherapy. Recently, the combination of venetoclax plus a hypomethylating agent (HMA; azacitidine or decitabine) or low‐dose cytarabine (LDAC) showed promise in Phase III trials in previously untreated AML.^2^, ^3^ DiNardo et al^2^ reported median overall survival (OS) of 14.7 months for venetoclax‐azacitidine versus 9.6 months for placebo‐azacitidine. Wei et al^3^ reported median OS of 8.4 months for venetoclax‐LDAC versus 4.1 months for placebo‐LDAC. In France at the time of this study, venetoclax was not yet approved for AML and there were therefore no formal usage recommendations. Here we report the first study in a French cohort that assessed venetoclax in combination with existing treatments for AML under real‐life conditions.

## 2 MATERIALS AND METHODS

This retrospective, observational study included patients with AML (World Health Organization 2016 classification)^4^ who were ineligible for intensive chemotherapy and received venetoclax, from 14 French centers, between February 2017 and May 2020. The study was conducted in compliance with reference methodology MR004 (V3.1 23/01/2020) and all patients gave informed consent.

The primary objective was to collect pseudonymized data on venetoclax use and management. We also investigated safety and tolerability (National Cancer Institute Common Terminology criteria for Adverse Events, version 4.03), progression‐free survival (PFS, European Leukemia Network [ELN] 2017 criteria^5^), response (International Working Group response criteria for AML^6^), and pharmacist interactions. Cytogenetic risk categorization used ELN 2017 criteria.^5^

Statistical analyses used R software (v3.6.1; see Appendix S1). For descriptive data expressed as %, missing data were subtracted from the denominator (n/N is given where this applies).

## 3 RESULTS

Data were collected from 118 patients with AML (67.8% de novo, 32.2% secondary) (Table 1; Table S1). Median follow‐up was 3.2 months (range 0.1–25.8).

**TABLE 1:** Venetoclax use and management

	Total cohort	Venetoclax‐HMA	Venetoclax‐LDAC	*P* value (HMA vs. LDAC)
No. of patients (% of total cohort)	118	91 (77.1) Azacitidine 89, decitabine 2	22 (18.6)	
First line or relapsed/refractory, *n*/*N* (%)				
First line	37/118 (31.4)	33/91 (36.3)	3/22 (13.6)	
Second line	57/118 (48.3)	41/91 (45.1)	13/22 (59.1)	‐
Third line or beyond	24/118 (20.3)	17/91 (18.7)	6/22 (27.3)	
Median duration of treatment (range)	2.5 months (0.03–16.2)	2.3 months (0.1–16.2)	3.1 months (0.3–9.2)	0.913
Complete cycles, *n*/*N* (%)				
Median (range)	2 (0–18)	2 (0–18)	2 (0–10)	0.105
1–3	70/117 (59.8)	59/91 (64.8)	10/21 (47.6)	
4–9	32/117 (27.3)	22/91 (24.2)	6/21 (28.6)	
≥10	4/117 (3.4)	3/91 (3.3)	1/21 (4.8)	
Dosage adjustment at initiation, *n*/*N* (%)^a^	59/117 (50.4)	42/91 (46.2)	15/21 (71.4)	0.065
For safety	10/59 (16.9)	6/42 (14.3)	2/15 (13.3)	
For drug interaction	49/59 (83.1)	36/42 (85.7)	13/15 (86.7)	
Dosage adjustment after >1 cycle, *n*/*N* (%)^a^ ^,^ ^b^	49/118 (41.5)	‐	‐	‐
For safety	35/49 (71.4)			
Venetoclax dose	22/35 (62.9)			
HMA/LDAC dose	9/35 (25.7)			
Schedule of treatment	10/35 (28.6)			
Treatment time interval	1/35 (2.9)			
For drug interaction	12/49 (24.5)			
Reasons for discontinuation, *n*/*N* (%)^c^				
Progressive disease	46/90 (51.1)	31/67 (46.3)	12/18 (66.7)	0.467
Toxicity	19/90 (21.1)	17/67 (25.4)	1/18 (5.6)	
Death	17/90 (18.9)	7/67 (10.4)	1/18 (5.6)	
Stem cell transplant	8/90 (8.9)	12/67 (17.9)	4/18 (22.2)	
Hematopoietic growth factor use, *n/N* (%)	26/115 (22.6)	25/88 (28.4)	1/22 (4.5)	0.022

*Note*: All denominators are indicated for individual values; missing data are subtracted from the denominator.

Abbreviations: HMA, hypomethylating agent; LDAC, low‐dose cytarabine.

^a^ Denominator for types of dosage adjustment is patients with any dosage adjustment.

^b^ Denominator for types of safety dosage adjustment is patients with any safety dosage adjustment.

^c^ Denominator for reasons for discontinuation is patients with any discontinuation.

The venetoclax‐based AML treatment was during relapse for 81 patients (68.6%), as second line in 48.3% and third line/beyond in 20.3%. For second‐line/beyond treatment, alongside venetoclax, 71.6% of patients received an HMA and 23.5% received LDAC. The venetoclax‐based AML treatment was first line in only 37 patients (31.4%). Overall, 28.8% of patients had prior HMA exposure, 33.1% had prior hydroxyurea exposure, and 26.3% had undergone allogeneic hematopoietic stem cell transplantation.

Median age at venetoclax initiation was 67 years (range 19–90) and 56.8% of patients were male. Comorbidity was present in 68.6% of patients, most commonly in cardiac disease. Of patients with cytogenetic risk categorization, 42.3% (44/104) exhibited poor risk and 51.9% (54/104) intermediate risk. Baseline *NPM1/2*, *FLT3*, *TP53*, and *IDH1/2* mutations were detected in 20.7% (23/111), 17.1% (19/111), 13.5% (15/111), and 4.5% (5/111) of patients, respectively.

Median venetoclax duration was 2.5 months (range 0.03–16.2), with a median of 2 (range 0–18) completed cycles. Most patients (89.0%) continued venetoclax into the second cycle; 12 discontinued venetoclax during the first 14–28 days and 1 received venetoclax for 21 days. The treatment discontinuation rate prior to the second cycle was 27.3% with venetoclax‐LDAC and 7.7% with venetoclax‐HMA (*p* = 0.027). For venetoclax initiation, it is standard practice to have a ramp‐up phase, but in this retrospective study, only 57.3% (67/117) of patients had a ramp‐up phase. A total of 74.6% were hospitalized (median 3 days [range 0–79]). The final venetoclax dose was significantly different between venetoclax‐HMA and venetoclax‐LDAC (*p* < 0.001), although most commonly for each were 100 mg/day (31.9% and 36.4% of patients, respectively) and 400 mg/day (52.7% and 22.7%, respectively).

Antifungal prophylaxis was used in 68.1% (79/116) of patients, most commonly azoles. Patients receiving antifungal prophylaxis had a higher rate of dosage adjustment at initiation due to drug interaction (*p* = 0.031) and a lower final venetoclax dose (*p* < 0.001) than those without antifungal prophylaxis. For initiation, the dosage was adjusted in 50.4% (59/117) of patients, due to drug interaction (*n* = 49, 43 receiving antifungal prophylaxis) and safety (*n* = 10). After the first cycle, dosage was adjusted in 41.5% of patients, due to safety (*n* = 35), drug interaction (*n* = 12, 8 receiving antifungal prophylaxis), and being in the COVID‐19 setting (*n* = 1). At the time of analysis, 22.0% of patients were still receiving venetoclax. Of 90 patients who discontinued venetoclax, primary reasons were progressive disease (disease progression or relapse; 51.1% of discontinuations), toxicity (21.1%), death (18.9%), and undergoing stem cell transplantation (8.9%).

Overall, 94.9% of patients experienced at least one treatment‐emergent grade 3/4 adverse event (Table S2). With all treatment lines and regimens, most common grade 3/4 adverse events were hematologic (overall 96.4% [108/112] of patients), infections (57.1% [64/112]), febrile neutropenia (37.5% [42/112]), and fatigue (31.3% [35/112]). Clinical tumor lysis syndrome (TLS) was reported in 11.9% of patients. Patients with TLS were significantly more likely to have initiated venetoclax without a ramp‐up phase than those without TLS (85.7% [12/14] vs. 53.4% [55/103], *p* = 0.040). Most common hematologic toxicities of any grade (Table S3) were neutropenia, thrombocytopenia, and anemia. Dosage adjustments for predominantly hematologic toxicity included adjusting the venetoclax dose and modifying the schedule. Red blood cell and platelet transfusions occurred in 87.3% and 76.3% of patients, respectively. Antibiotics were used in 77.1% of patients. Hematopoietic growth factors, such as granulocyte colony‐stimulating factor, were used in 22.6% (26/115) of patients.

For second‐line/beyond treatment, 77 patients were evaluable for efficacy outcomes by regimen (*n* = 58 venetoclax‐HMA, *n* = 19 venetoclax‐LDAC; Table 2; Figure S1). Median PFS was 4.0 months (95% confidence interval [CI] 2.7–12.8) with venetoclax‐HMA and 3.4 months (95% CI 1.3–8.9) with venetoclax‐LDAC. Overall response rate (ORR) was 51.9% (28/54) with venetoclax‐HMA and 41.2% (7/17) with venetoclax‐LDAC, including a complete response (CR) or CR with incomplete hematologic recovery (CRi) rate of 33.3% (18/54) and 23.5% (4/17), respectively. Median time to first response was 43 days (range 21–84) with venetoclax‐HMA and 28 days (range 28–112) with venetoclax‐LDAC, meaning most responses were between the first and second cycles; median response duration was 79.5 days (range 32–300) and 112 days (range 85–252), respectively. Cumulative incidence of relapse for second‐line/beyond treatment was 51.9% (95% CI 32.5–71.3) with venetoclax‐HMA and 63.5% (95% CI 38.1–89.0) with venetoclax‐LDAC (difference *p* = 0.268) (Figure S2).

**TABLE 2:** Efficacy outcomes

	Total cohort	First‐line treatment		Second‐line/beyond treatment	
		Venetoclax‐HMA	Venetoclax‐LDAC	Venetoclax‐HMA	Venetoclax‐LDAC
No. of patients	118	33	3	58	19
Treatment response, n/N (%)	65/115 (56.5)	24/32 (75.0)	1/3 (33.33)	28/55 (50.9)	7/18 (38.9)
Type of response, n/N (%)					
CR	25/113 (22.1)	11/32 (34.4)	0/3 (0)	11/54 (20.4)	2/17 (11.8)
CRi	12/113 (10.6)	3/32 (9.4)	0/3 (0)	7/54 (13.0)	2/17 (11.8)
PR	27/113 (23.9)	10/32 (31.3)	1/3 (33.3)	10/54 (18.5)	3/17 (17.6)
No response	49/113 (43.4)	8/32 (25.0)	2/3 (66.7)	26/54 (48.1)	10/17 (58.8)
Transfusion response, n/N (%)^a^					
Decrease in transfusion requirement	46/107 (43.0)	17/31 (54.8)	1/3 (33.3)	18/50 (36.0)	7/18 (38.9)
No response	61/107 (57.0)	14/31 (45.2)	2/3 (66.7)	32/50 (64.0)	11/18 (61.1)
Median time to first response (range)	30 days (7–168)	28 days (7–168)	42 days (NA)	43 days (21–84)	28 days (28–112)
Median duration of response (range)	94.5 days (15–504)	86.5 days (15–504)	NA	79.5 days (32–300)	112 days (84–252)
Median progression‐free survival (95% CI)	3.9 months (3.1–5.6)	5.3 months (2.3‐NR)	NR months (0.9‐NR)	4.0 months (2.7–12.8)	3.4 months (1.3–8.9)

*Note*: All denominators are indicated for individual values; missing data are subtracted from the denominator.

Abbreviations: CI, confidence interval; CR, complete response; CRi, CR with incomplete hematologic recovery; HMA, hypomethylating agent; LDAC, low‐dose cytarabine; NA, not applicable; NR, not reached; PR, partial response.

^a^ An observed reduction in need for transfusion or reduction in delay between two transfusions.

For first‐line treatment, 36 patients were evaluable for efficacy outcomes, the majority of whom received venetoclax‐HMA (*n* = 33). With first‐line venetoclax‐HMA, median PFS was 5.3 months (95% CI 2.3–not reached), ORR was 75.0% (24/32), CR/CRi was 43.8% (14/32), median time to response was 28 days (range 7–168), and median response duration was 86.5 days (range 15–504).

Multivariate analysis showed independent associations for lower ORR versus Eastern Cooperative Oncology Group performance status 2 (0.27, 0.10–0.78, *p* = 0.015), second‐ or third‐line treatment (0.47, 0.25–0.81, *p* = 0.02 and 0.51, 0.25–1.01, *p* = 0.05, respectively), and previous solid tumor history (0.31, 0.12–0.77, *p* = 0.01). These associations should though be interpreted with caution due to the different treatment settings, treatment lines, and regimens.

An interview with a clinical pharmacist was held for 17.8% of patients. Communication between hospital and ambulatory medicine occurred for 40.9% (45/110) of patients. A medical reconciliation, where patient's treatments were compiled and cross‐referenced with several information sources, was undertaken for 29.7% of patients. A pharmacist intervention was conducted for 13.6% of patients, mostly for drug interactions.

## 4 DISCUSSION

Our observations around the real‐life use, management, and safety of venetoclax in patients with AML in French centers were in line with previous findings.^2^, ^3^, ^7^, ^8^, ^9^ Since venetoclax is metabolized by CYP3A4, dosage adjustments for drug interactions were expected,^10^, ^11^, ^12^ including when using azole CYP3A4 inhibitors for antifungal prophylaxis.^10^ Rates of cytopenia were similar to those in other real‐life studies.^8^, ^9^ Of note, we found that contrary to standard practice a ramp‐up phase was not always used for venetoclax initiation, and that incompliance with a ramp‐up phase increased the likelihood of TLS. We also observed variations between our centers in dosage adjustments made both for drug interactions and safety. Algorithms to address dosage adjustments are now present in recommendations for venetoclax‐based treatment in patients with AML,^13^, ^14^, ^15^ which may help to harmonize practices.

Our heterogeneous real‐life population makes comparisons with the Phase III trials difficult, with no restrictions on treatment lines, prior hematologic disorders, prior HMA and hydroxyurea exposure, and cytogenetic risk groups. Moreover, our sample size is limited, especially the first‐line cohort of only 37 patients. Nonetheless, our findings for venetoclax‐based treatment in patients ineligible for intensive chemotherapy were promising, including in relapsed/refractory AML and when combined with an HMA. Our second‐line/beyond ORR of 51.9% with venetoclax‐HMA and 41.2% with venetoclax‐LDAC, and CR/CRi rate of 33.3% and 23.5%, respectively, were comparable to those in other studies.^8^, ^9^, ^16^, ^17^, ^18^ However, our first‐line CR/CRi rate of 43.8% with venetoclax‐HMA was lower than in a Phase III trial (66%)^2^ and our first‐line median response duration of 105 days was also relatively short (17.8 months in Phase III trial).

Our findings showed evidence of a multidisciplinary approach, although this was not consistent. Clinical pharmacists play key roles in venetoclax management, including patient education, building links between hospital and ambulatory medicine, and collaboration around drug interactions. First developed in France for lymphoid disease, ambulatory medical assistance (AMA) is a multidisciplinary program that uses phone calls from nurses to monitor patients who are undergoing active home treatment.^19^ During active‐phase treatment in patients with hematologic malignancies, the AMA program was demonstrated to ensure safe care through avoidance of adverse events (hematologic or nonhematologic).^20^ Patients with AML treated with new molecules are thus good candidates for an AMA program.

In conclusion, this real‐life study in France showed that venetoclax‐based treatment yields promising findings in patients with AML ineligible for intensive chemotherapy, but its use and management are complex, particularly in terms of dosage adjustments. Practice harmonization, along with greater clinical pharmacist and nurse involvement, could potentially be future improvements.

## AUTHOR CONTRIBUTIONS

**Louise Laloi:** Conceptualization (lead); formal analysis (lead); investigation (lead); writing – original draft (lead); writing – review and editing (lead). **Natacha Chaumard Billotey:** Formal analysis (lead); methodology (lead); supervision (lead); validation (lead); writing – original draft (lead); writing – review and editing (lead). **Pierre‐Yves Dumas:** Resources (equal); validation (equal). **Franciane Paul:** Resources (equal); validation (equal). **Alban Villate:** Resources (equal); validation (equal). **Célestine Simand:** Resources (equal); validation (equal). **Luc Fornecker:** Resources (equal); validation (equal). **Florent Puisset:** Resources (equal); validation (equal). **Sarah Bertoli:** Resources (equal); validation (equal). **Marion Boissard Simonet:** Resources (equal); validation (equal). **Kamel Laribi:** Resources (equal); validation (equal). **Dyhia Houyou:** Resources (equal); validation (equal). **Alberto Santagostino:** Resources (equal); validation (equal). **Claire Michel:** Resources (equal); validation (equal). **Gabrielle Roth Guepin:** Resources (equal); validation (equal). **Elodie Guerineau:** Resources (equal); validation (equal). **Reza Tabrizi:** Resources (equal); validation (equal). **Mathilde Hunault:** Resources (equal); validation (equal). **Aurélien Giltat:** Resources (equal); validation (equal). **Eléonore Kaphan:** Resources (equal); validation (equal). **Claude Bulabois:** Resources (equal); validation (equal). **Elodie Cartet:** Resources (equal); validation (equal). **Clément Rocher:** Resources (equal); validation (equal). **Florence Lachenal:** Resources (equal); validation (equal). **Stéphane Morisset:** Methodology (lead); resources (lead); software (lead); supervision (lead); validation (lead). **Christian Récher:** Resources (equal); supervision (equal); validation (equal); visualization (equal); writing – original draft (equal); writing – review and editing (equal). **Arnaud Pigneux:** Resources (equal); validation (equal); visualization (equal); writing – original draft (equal); writing – review and editing (equal). **Amine Belhabri:** Resources (equal); supervision (equal); visualization (equal). **Mauricette Michallet:** Supervision (equal); validation (equal); visualization (equal). **Anne‐Sophie Michallet:** Conceptualization (lead); formal analysis (lead); investigation (lead); methodology (lead); resources (lead); supervision (equal); validation (equal); writing – original draft (lead); writing – review and editing (lead).

## FUNDING INFORMATION

Not applicable.

## CONFLICT OF INTEREST

The authors declare that they have no conflict of interest.

## ETHICAL APPROVAL

The study was approved by the French Innovative Leukemia Organization (FILO) Scientific Committee.

## PATIENT CONSENT

Not applicable.

## Supporting information


Appendix S1

## Data Availability

Data available on request from the authors.
