# Supplementary material for: Retrospective, real‐life study of venetoclax plus azacitidine or low‐dose cytarabine in French patients with acute myeloid leukemia ineligible for intensive chemotherapy
Source: Cancer Med. 2022 Dec 8;12(6):7175–81. doi: 10.1002/cam4.5459 (PMC10067034; doi:10.1002/cam4.5459)

**Retrospective, real-life study of venetoclax plus azacitidine or low-dose cytarabine in French patients with acute myeloid leukemia ineligible for intensive chemotherapy**

**Supporting information**

**Statistical analyses**

All statistical analyses were performed using R software (v3.6.1). Analyses were for the total cohort; some descriptive analyses (patient baseline characteristics, venetoclax treatment management) and all efficacy analyses were also by combination with a hypomethylating agent (HMA, azacitidine or decitabine) or low-dose cytarabine (LDAC). Qualitative variables were expressed as % (n/N), with missing data subtracted from the denominator. Descriptive group comparisons for normally distributed groups used Student’s t-test (2 groups) or ANOVA test (>2 groups). Otherwise, non-parametric tests (Mann-Whitney or Kruskal-Wallis) were applied. Quantitative variables were expressed as median (range). Pearson’s Chi-squared test was performed for group comparisons (Montecarlo simulation applied if any count was <5).

Treatment response used the cumulative incidence function (with death without progression as competing risk), with the Gray test applied. Progression-free survival curves since treatment initiation used Kaplan-Meier method, with the log-rank test applied.

Risk factors versus outcomes were analyzed by logistic regression or by Fine and Gray regression model (with death without progression a competing risk). Following univariate analyses, variables presenting with *P*<5% and over 80% data availability were implemented into a multivariate model. The two-sided significance level was 5% and estimations were presented with 95% confidence intervals.

**Table S1.** Patient baseline demographics and clinical characteristics

|  | **Total cohort** | **Venetoclax-HMA^a^** | **Venetoclax-LDAC** |
| --- | --- | --- | --- |
| No. of patients^b^ | 118 | 91 | 22 |
| Male gender, n/N (%) | 67/118 (56.8) | 50/91 (54.9) | 14/22 (63.6) |
| Median age at AML diagnosis (range) | 65.5 years (18-89) | 63 years (20-89) | 69 years (18-84) |
| Median age at venetoclax initiation (range) | 67 years (19-90) | 65 years (20-90) | 70 years (19-84) |
| ECOG performance status score, n/N (%)  0-1  2-4 | 74/111 (66.7) 37/111 (33.3) | 56/84 (66.7) 28/84 (33.3) | 15/22 (68.2) 7/22 (31.8) |
| AML type, n/N (%)  *De novo*  Secondary | 80/118 (67.8) 38/118 (32.2) | 65/91 (71.4) 26/91 (28.6) | 14/22 (63.6) 8/22 (36.4) |
| Bone marrow blast count at initiation, n/N (%)  <30%  30-50%  >50% | 50/111 (45.0) 23/111 (20.7) 38/111 (34.2) | 40/85 (47.1) 18/85 (21.2) 27/85 (31.8) | 8/21(38.1) 5/21 (23.8) 8/21 (38.1) |
| Comorbidities, n/N (%)^c^  Cardiac disease  Solid tumor  Blood disease  Dyslipidemia  Thyroid disease  Diabetes  Obesity  Transient ischemic attack/stroke  Chronic obstructive pulmonary disease  Chronic renal failure  Other | 81/118 (68.6) 53/80 (66.3) 18/80 (22.5) 16/80 (20.0) 12/80 (15.0) 11/80 (13.8) 10/80 (12.5) 10/80 (12.5) 7/80 (8.8) 6/80 (7.5) 4/80 (5.0) 18/80 (22.5) | 62/91 (68.1) | 15/22 (68.2) |
| Cytogenetic risk category, n/N (%)  Low  Medium  Poor | 6/104 (5.8) 54/104 (51.9) 44/104 (42.3) | 5/79 (6.3) 38/79 (48.1) 36/79 (45.6) | 1/20 (5.0) 13/20 (65.0) 6/20 (30.0) |

AML: acute myeloid leukemia; ECOG: Eastern Cooperative Oncology Group;
HMA: hypomethylating agent; LDAC: low-dose cytarabine

^a^Azacitidine n=89, decitabine n=2
^b^All denominators are indicated for individual values; missing data are subtracted from the denominator
^c^Denominator for types of comorbidity is patients with any comorbidity

**Table S2.** Grade 3/4 adverse events

| **Event, n/N (%)^a,b^** | **Total cohort (N=118)** | **First-line  treatment** | | **Second-line/beyond treatment** | |
| --- | --- | --- | --- | --- | --- |
|  |  | **Venetoclax-HMA** | **Venetoclax-LDAC** | **Venetoclax-HMA** | **Venetoclax-LDAC** |
| No. of patients | 118 | 33 | 3 | 58 | 19 |
| Any grade 3/4 adverse event | 112/118 (94.9) | 32/33 (97.0) | 2/3 (66.7) | 54/58 (93.1) | 19/19 (100) |
| Hematologic toxicity | 108/112 (96.4) | 31/31 (100) | 3/3 (100) | 50/54 (92.6) | 19/19 (100) |
| Infections | 64/112 (57.1) | 14/31 (45.2) | 3/3 (100) | 37/54 (68.5) | 9/19 (47.4) |
| Febrile neutropenia | 42/112 (37.5) | 12/31 (38.7) | 1/3 (33.3) | 19/54 (35.2) | 8/19 (42.1) |
| Impaired general condition – fatigue | 35/112 (31.3) | 13/31 (41.9) | 0/3 (0) | 14/54 (25.9) | 5/19 (26.3) |
| Digestive disorders  Diarrhea  Nausea and/or vomiting  Constipation  Gastro-esophageal reflux – esophagitis | 33/112 (29.5) 11/112 (9.8) 6/112 (5.4) 3/112 (2.7) 1/112 (0.9) | 5/31 (16.1)  3/31 (9.7)  1/31 (3.2)  1/31 (3.2)  0/31 (0) | 0/3 (0)  0/3 (0)  0/3 (0)  0/3 (0)  0/3 (0) | 12/54 (22.2)  8/54 (14.8)  2/54 (3.7)  2/54 (3.7)  0/54 (0) | 3/19 (15.8)  0/19 (0)  2/19 (10.5)  0/19 (0)  1/19 (5.3) |
| Hypokalemia | 7/112 (6.3) | 2/31 (6.5) | 0/3 (0) | 3/54 (5.6) | 2/19 (10.5) |
| Decreased appetite –  weight loss | 6/112 (5.4) | 1/31 (3.2) | 0/3 (0) | 4/54 (7.4) | 0/19 (0) |
| Cardiac disorders | 3/112 (2.7) | 1/31 (3.2) | 0/3 (0) | 2/54 (3.7) | 0/19 (0) |

^a^Denominator for types of grade 3/4 adverse event is patients with any grade 3/4 adverse event
^b^Others: renal toxicity (n=5), hepatic disorder (n=4), edema (n=3), mucositis (n=3), muscular pain (n=2), stroke (n=2), swallowing disorder (n=2), acute pulmonary edema (n=1), hyperuricemia (n=1), and neuropathy (n=1)

**Table S3.** Hematologic toxicity and dosage adjustments, by grade

| **Event, n/N (%)^a,b^** | **Total cohort (N=118)** | **Dose interruption** | **Dose reduction** | **Permanent discontinu- ation** | **Increased treatment time interval** | **Supportive treatment** |
| --- | --- | --- | --- | --- | --- | --- |
| Neutropenia  Grade 0-1  Grade 2  Grade 3^c^  Grade 4^d^ | 109/118 (92.4) - 4/104 (3.8) 23/104 (22.1) 77/104 (74.0) | 21/109 (19.3) - - 4 17 | 14/109 (12.8) - 1 4 9 | 10/109 (9.2) - - 3 7 | 8/109 (7.3) - - 3 5 | 71/109 (65.1) - 3 12 55 |
| Thrombo-cytopenia  Grade 0-1  Grade 2  Grade 3^e^  Grade 4^f^ | 100/117 (85.5)  5/95(5.3) 8/95 (8.4) 16/95 (16.8) 66/95 (69.5) | 3/100 (3.0)  - - 1 2 | 7/100 (7.0)  - 1 3 3 | 4/100 (4.0)  - - 1 2 | 2/100 (2.0)  - - 2 1 | 83/100 (83.0)  3 7 11 60 |
| Anemia  Grade 0-1  Grade 2^g^  Grade 3  Grade 4^h^ | 111/118 (94.1) 7/104 (6.7) 25/104 (24.0) 50/104 (48.1) 22/104 (21.2) | 2/111 (1.8) - 1 1 - | 6/111 (5.4) - 5 1 - | 9/111 (8.1) - 3 1 5 | 1/111 (0.9) - 1 - - | 87/111 (78.4) 5 15 47 17 |

^a^All denominators are indicated for individual values; missing data are subtracted from the denominator
^b^Denominators for grades of a hematologic toxicity, and for types of dosage adjustment, are patients with the hematologic toxicity

Included patients with >1 dosage adjustment type: ^c^2 associated, ^d^17 associated, ^e^1 associated, ^f^2 associated, ^g^1 associated, ^h^1 associated

**Figure S1. Progression-free survival (PFS) according to venetoclax treatment line and concomitant treatment with a hypomethylating agent (HMA) or low-dose cytarabine (LDAC)**


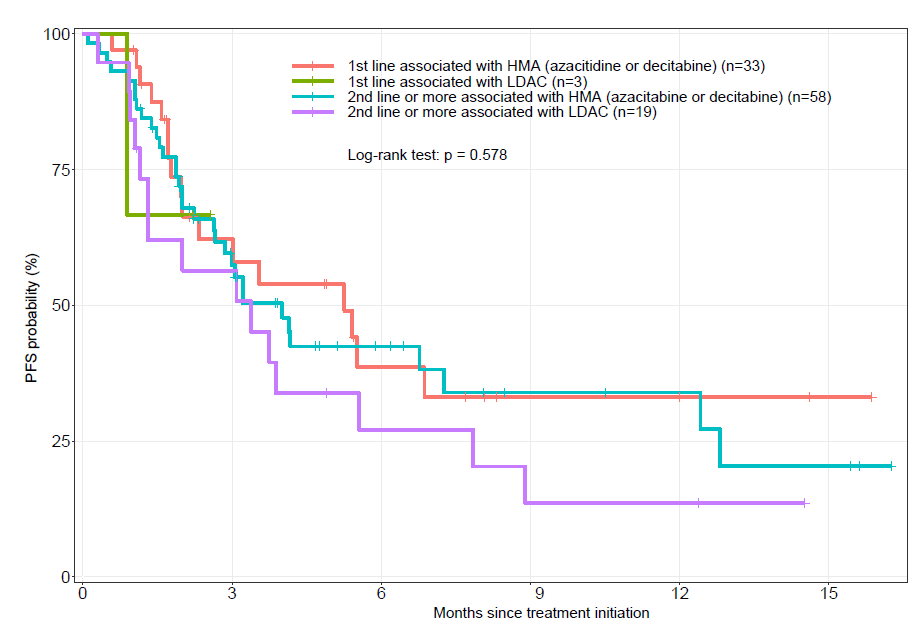


**Figure S2. Cumulative incidence of relapse according to venetoclax treatment line and concomitant treatment with a hypomethylating agent (HMA) or low-dose cytarabine (LDAC)**


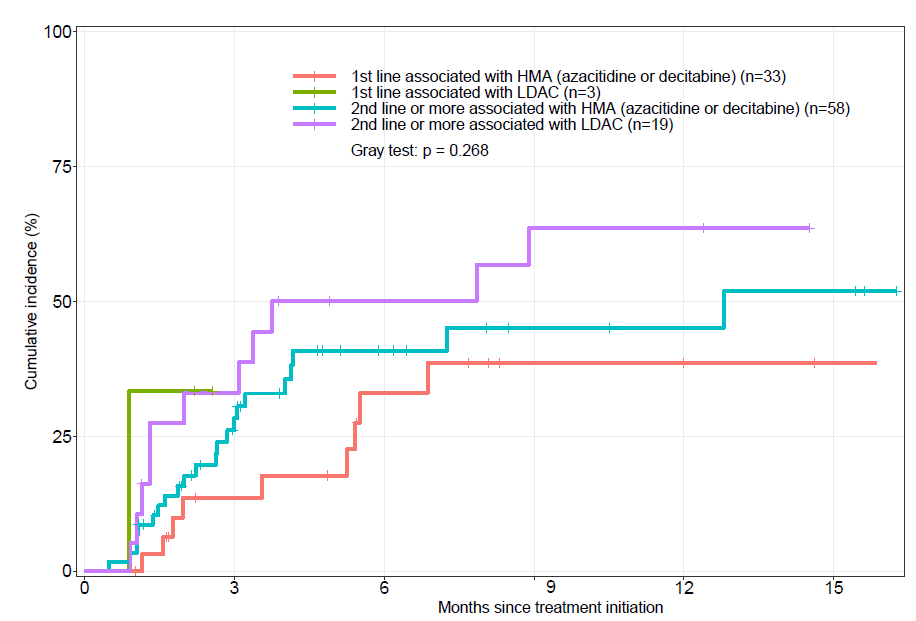

Supplement: Supplementary file 1 — Appendix S1 [file CAM4-12-7175-s001.docx]
